# Shilling attack detection for recommender systems based on credibility of group users and rating time series

**DOI:** 10.1371/journal.pone.0196533

**Published:** 2018-05-09

**Authors:** Wei Zhou, Junhao Wen, Qiang Qu, Jun Zeng, Tian Cheng

**Affiliations:** 1 Shenzhen Institutes of Advanced Technology, Chinese Academy of Sciences, Shenzhen, China, 518055; 2 School of Big Data & Software Engineering, Chongqing University, Chongqing, China, 40044; Victoria University, AUSTRALIA

## Abstract

Recommender systems are vulnerable to shilling attacks. Forged user-generated content data, such as user ratings and reviews, are used by attackers to manipulate recommendation rankings. Shilling attack detection in recommender systems is of great significance to maintain the fairness and sustainability of recommender systems. The current studies have problems in terms of the poor universality of algorithms, difficulty in selection of user profile attributes, and lack of an optimization mechanism. In this paper, a shilling behaviour detection structure based on abnormal group user findings and rating time series analysis is proposed. This paper adds to the current understanding in the field by studying the credibility evaluation model in-depth based on the rating prediction model to derive proximity-based predictions. A method for detecting suspicious ratings based on suspicious time windows and target item analysis is proposed. Suspicious rating time segments are determined by constructing a time series, and data streams of the rating items are examined and suspicious rating segments are checked. To analyse features of shilling attacks by a group user’s credibility, an abnormal group user discovery method based on time series and time window is proposed. Standard testing datasets are used to verify the effect of the proposed method.

## 1 Introduction

With the development of e-commerce, information overload is a serious problem [1]. As a kind of technology to generate recommendations by establishing a binary relationship between users and items, recommendation systems can alleviate the information overload problem effectively and have thus become a solution in information retrieval area. Recommender systems can be divided into two categories, collaborative filtering and content-based filtering. Recommendation systems are widely used in many fields, such as movie recommendations (Netflix), news recommendations (Toutiao), book recommendations (Amazon), image recommendations (Flickr), music recommendations (Last.fm), restaurant recommendations (TripAdvisor), and video recommendations (Youtube).

However, with the increment of the number of users and items in recommendation systems (online websites), more *UGC* data are generated [2]. Many new features are introduced with the development of recommender systems, which presents new challenges. Recommender systems are extremely vulnerable to attack, and so this problem needs to be urgently addressed. Accurate recommendations and a good online user experience can help e-commerce sites to have more success over their competition. Therefore, online retailers try to make their own products rank at the top of recommendation lists, which will increase their sales and profits. However, for economic motives, malicious users inject forged *UGC* profiles to affect the recommendation list of recommender systems [3, 4]. Some attacks may try to “push” targeted items (push attacks), while others may aim to “nuke” some targeted items (nuke attacks). Attackers manipulate recommendation frequency of target items by falsifying user profiles [5, 6]. Sony Pictures uses counterfeit film reviews to recommend new releases to users [7]. According to the United States Cone Company survey, 64% of users make a purchase before referencing existing user comments.

Some users inject fake user profiles consisting of biased ratings to affect the recommendation ranking and manipulate the user’s decision. Attacks on recommender system behaviour is known as a “shilling” attack or “profile injection” attack. Users that carry out shilling attacks are known as attackers or shillers. Push attacks and nuke attacks are the two most common types of attacks. Recommendation lists of specified items are affected based on the intent of the attacker. In push attacks, attackers try to make the specified items be recommended more by injecting biased ratings, whereas nuke attacks occur in the opposite way. Thus, detecting shilling attack profiles and eliminating adverse effects are the best methods to maintain the robustness of recommender systems.

Causing their own products to be among the top rankings means money. In some situation, a group of attackers can quickly push a particular item to a referral list [8]. Malicious users use faked identities to create user profiles and then manipulate the recommendation list of a specific target item Injecting attack profiles to recommender system costs time and other resources. Attackers should consider the benefit/cost ratio when they perform shilling attacks. Studies show that group attack profiles are used to manipulate recommendation ranking of target items. Many attackers work together to perform an attack on specific target items in a particular time frame.

Shilling attacks affect all participants involved. Shilling attacks can cause a lot of damage in user-based recommender systems. There are 3 kinds of participants in the recommendation process: users (genuine users and attackers), items (movies, videos, books) and the recommender system itself. Shilling attacks result in unfair competition, resulting in loss of normal users and recommender systems [5]. Normal users cannot select the most wanted items because of the shilling attacks. Shilling attacks affect the recommendation list, which will reduce the reputation of the recommendation platform. To make improve recommendation technologies and increase fairness among users, it is very important to develop detection technology.

In this paper, a shilling detection method based on the credibility of group users and rating time series is proposed. Considering the group and timeliness features of shilling attacks, a credibility evaluation method of group users based on the rating prediction model is also proposed. We propose a method of detecting suspicious ratings based on target item analysis and rating time series. By constructing a time series to determine the suspect rating time intervals, the constructed data stream is then examined and the suspect rating segment is examined. Based on the analysis of the characteristics of a group user’s attacks, a method to discover abnormal group users based on time series and time windows is proposed. We tested the method on different datasets to verify the model and algorithm. Experiments show that this method performs well in the detection of a large dataset of shilling attacks.

## 2 Related work

Malicious users, also known as shilling attackers, inject forged *UGC* data into recommender systems to manipulate recommendation rankings of recommender systems; this behaviour is known as a profile injection attack [9]. Studies [10, 11] show that collaborative filtering recommendation systems, whether user-based or project-based, are vulnerable to profile attacks. Attackers forge rating profiles and inject them into the rating matrix of recommender systems. To make attack profiles more difficult to detect, attackers rate both target and non-target items according to the attack type, which makes attack profiles look like normal ones.

Shilling attack detection can be considered as a two-classification problem [12]. Attack classifiers can be achieved through classification or clustering techniques. User profile properties are extracted using machine learning methods to detect attack profiles. Shilling detection method can be categorized into several categories by the number of labelled tags: supervised, semi-supervised and unsupervised methods [13].

A number of studies have employed supervised method to detect shilling profiles [14, 15]. In order to train a model, labelled data is used in these supervised methods, and the quality of labelled data influence the detecting result directly. Features of attack profiles are extracted and supervised detecting method is built on these features. These methods only consider individual user’s features but ignore the relationship between attack profiles. Moreover, these supervised methods do not perform well in blurring attack profile detection. Unsupervised based methods address shilling attack issues by training unlabelled datasets [16, 17]. Some assumptions and priori knowledge are needed before perform these methods. These methods involve much less computation than supervised methods. The benefit of doing this is that these methods can be used in online detection. Some techniques use clustering, association rule methods, and statistical methods.

Semi-supervised based methods [18, 19] use both unlabelled and labelled profiles. Semi-supervised learning is a learning technique between supervised learning and unsupervised learning, which learns both tagged and untagged data. It is hard to get enough labelled data, semi-supervised based methods perform well with less labelled data than supervise based methods. Shilling attack models will evolve with changes in shilling detection methods. Once attackers are aware of shilling detection mechanisms, they will react quickly reduce the effectiveness of the detection method. Thus, there is a downside to fixed shilling attack methods. In this paper, all ratings for each item are sorted by time stamps; abnormal rating segments are determined by examining the time stamps. Statistical measures and target item analysis methods were used to detect shilling attacks.

## 3 Problem definition

In this section, in order to better understand shilling attacks and detection methods, some basic concepts are first defined and used. Secondly, we introduce the common measures of shilling attacks and several kinds of shilling models. Finally, the concept of group user trust model and time series are introduced.

### 3.1 Definitions

**Fig 1:**
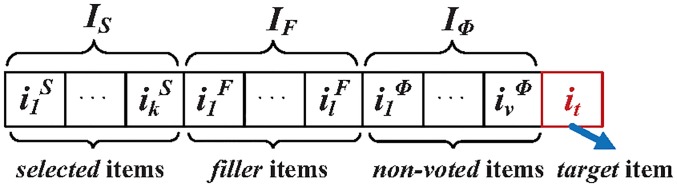
The general composition of an attack profile.

In this section, some basic definitions and concepts about shilling attacks are introduced, including several popular shilling attacks and definitions and analysis of some metrics. A user-item rating matrix is composed of three components, including users, items and ratings. We agree that:

$$\begin{array}{c} U=\{u_{1},u_{2},u_{3},\cdots u_{m-1},u_{m}\} \end{array}$$ (1)

where *U* is users set, *m* is the number of users. We set:

$$\begin{array}{c} I=\{I_{1},I_{2},I_{3},\cdots I_{n-1},I_{m}\} \end{array}$$ (2)

where *I* is all items set, *n* is the number of items. Rating time stamps *t* are the rating time stamp of a user rating for an item. User profile *P* are all ratings written by a user. If ratings in a profile are generated without disturbance, the profile is determined to be a genuine profile. Conversely, if a profile is forged, the profile is identified as an attack profile. User-item rating matrix *R* is the set of all user profiles, while *r*_*mn*_ is the rating score (from 1–5) from User *m* rates on Item *n*. For example, a rating of 5 indicates that User *m* likes Item *i*, while a rating of 1 indicates that User *m* dislikes Item *i*. If the user does not rate an item, *r*_*mn*_ is set to 0, and the relative item is marked as unrated. A user profile contains all rated (ranging from 1–5) and unrated items (with a rating of 0). An attack profile is a type of user profile with forged ratings. Attack profiles contain filler items, selected items, unrated items and target item(s); The selected items are determined by the attacker to form characteristics of the attack; For example, filler items are chosen randomly, but unrated items are also present. The target item is the item that an attacker attempts to push or nuke. The composition of an attack profile is shown in Fig 1.

To mask the attack profile as genuine and make it difficult to detect, attackers try to forge ratings to match those of genuine profiles. According to the composition of attack profiles, different attack model are defined. Details of attack models will be introduced later. Push and nuke attacks are two types based on the attackers’ intentions. Push attacks are aimed at boosting items and increasing their rankings, while nuke attacks are designed to downgrade items and reduce their rankings. In this paper, we mean push attacks if not specialized.

Generally speaking, in order to get a better cost/benefit ratio, attackers perform an attack using a group of users in a short period of time; Target items are assigned a high score (push attack) or low score (nuke attack), while filler items are assigned with forged scores according to the attack models used. Rating deviation of Attack profiles’ rating deviation is greater than genuine profiles’ rating deviation, the credibility of group users in some time interval based on rating deviation would be lower than that of normal profiles.

### 3.2 Shilling attack models

According to attackers’ knowledge and usage, four popular attack modes have been identified: random, average, bandwagon and segment attack models [14]. In general, there are 3 parts in a profile: target item(s) set *I*_*T*_; Selected set *I*_*S*_, attackers usually select some items that have similar characteristics with the target item(s); and filler set *I*_*F*_, which is a selection of items that make the attack profile be similar to normal ones. Table 1 shows the composition of each attack models.

**Table 1:** Features of attack models.

Attack Model	*I*_*S*_(Selected Set)	*I*_*F*_(Filler Set)	*I*_*T*_(Target Set)
**Random**	∅	random selected	*r*_*max*_/*r*_*min*_
**Average**	∅	mean of items	*r*_*max*_/*r*_*min*_
**Bandwagon**	*r*_*max*_	popular items	*r*_*max*_/*r*_*min*_
**Segment**	*r*_*max*_	segment items	*r*_*max*_/*r*_*min*_

The quality of attack profiles depends on prior knowledge attackers gained before they perform an attack. More prior knowledge is obtained, the attack profiles are more complex, the profiles appear more genuine. The main difference between attack models is the distribution of variance ratings for filler set and selected set.

Random attack uses a normal distribution to evaluate randomly choose filler items around the system’s average ratings, which is described in [20]. Highest or lowest score are assigned to target items according to attack types. The average attack requires priori knowledge of average rating of items in the recommender system. Normal distribution is used to randomly rate items with the average score set as the mean rating of items according to the standard deviation [20]. Attackers disguise themselves and are more difficult to distinguish than genuine users, thus giving them greater impact on recommendations. The target items’ ratings are the same like random attack models. Bandwagon and segment attack models are evaluated from random and average attack models [5]. These profiles select a lot of items of a certain kind, for example, popular or high rated items. Selected items in segment attacks and bandwagon attacks are group attacks. The rationale behind group attacks is attackers focus on those who are already inclined to the product, which can promote attack efficiency. In other words, an attacker intending to promote a particular item not to all users, but instead recommending it to potential users.

### 3.3 User-based collaborative filtering recommendation

Below is the process of generating a recommendation. A user-item matrix is constructed by collecting ratings of users on items in user-based collaborative filtering systems, *U*_*m*×*n*_, containing rating information from *n* users on *m* items. First, similar or dissimilar rules are established between users and then the most important users of similarities or dissimilarities are determined. Prediction is ultimately calculated by considering the user (or item) scores and their similarities. The prediction is calculated as weighted average of the *z*-scores, as follows:

$$\begin{array}{c} p_{at}=v_{a}+\sigma_{a}\times \frac{\sum_{u\in N}w_{au}\times z_{ut}}{\sum_{u\in N}w_{au}} \end{array}$$ (3)

where N is the set of neighbours of user a and *w*(*au*) is the similarity weight between User *a* and User *u*. The similarity can be calculated as

$$\begin{array}{c} w_{au}=\frac{\sum_{j\in M}\left(v_{aj}-\bar{v_{a}})\times (v_{uj}-\bar{v_{u}}\right)}{\sigma_{a}\times \sigma_{u}} \end{array}$$ (4)

where Set *M* is items rated by both User *a* and User *u*.

$$\begin{array}{c} z_{uj}=(v_{uj}-\bar{v_{u}})/\sigma_{u} \end{array}$$ (5)

where *z*_*uj*_ is the normalized ratings instead of actual ratings that User *u* rates for Item *j*. *v*_*uj*_ is the actual rating of User *u* for Item *j*. $\bar{v_{u}}$ is the mean rating of user *u* for all items, and *σ*_*u*_ is the standard deviation of the ratings for user *u*, respectively.

In the *K* nearest neighbours recommendation methods, the most similar *k* neighbours are selected. This algorithm tends to positively consider correlated neighbours only, recommendation rankings are sorted by the similarity values. The intent of attackers try to use the weakness of the recommendation methods, trying to manipulate the target item(s) in the recommendation list.

### 3.4 Prediction shift and rating variances of shilling attacks

The predicted shift is a measure of the changes of rating scores in the predicted value before and after the implementation of an attack. Prediction shift is usually used to measure the impact of an attack to recommender systems. *p*_*u*, *i*_ is prediction score of User *u* on Item *i* before an attack.

$$\begin{array}{c} p_{u,i}=\bar{r_{u}}+\frac{\sum {}_{v\in U_{u,i}}[w_{u,v}(r_{v,i}-\bar{r_{v}})]}{\sum {}_{v\in U_{u,i}}|w_{u,v}|} \end{array}$$ (6)

*p_u,i_*′ is the prediction score of User *u* on Item *i* after attack profiles are injected into the rating matrix; *p*_*u*,*i*_ is the prediction score of User *u* on Item *i* before attack profiles are injected into the rating matrix;

$$\begin{array}{c} {p_{u,i}}^{\prime }=\bar{r_{u}}+\frac{\sum {}_{v\in U_{u,i}}[w_{u,v}(r_{v,i}-\bar{r_{v}})]}{\sum {}_{v\in U_{u,i}}|w_{u,v}|} \end{array}$$ (7)

Δ_*u*,__*i*_ is the difference between *p*_*u*,*i*_ and *p_u,i_*′.

$$\begin{array}{c} {\Delta_{u,}}_{i}=|{p_{u,i}}^{\prime }-p_{u,i}| \end{array}$$ (8)

The prediction shift of User *u* on Item *i* can be calculated by the following equation:

$$\begin{array}{c} \Delta_{i}=\sum_{u\in U_{T}}\frac{\Delta_{u,i}}{\left|U_{T}\right|} \end{array}$$ (9)

The prediction shift of all items in the rating matrix after an attack is performed can be calculated by the following equation:

$$\begin{array}{c} \bar{\Delta }=\sum_{u\in I_{T}}\frac{\Delta_{i}}{I_{T}} \end{array}$$ (10)

To test the impact of shilling attacks on user-based recommended systems, an experiment was designed to calculate the prediction shift when different types of shilling attacks are injected into the rating matrix. In this experiment, the MovieLens ML100K dataset was used to calculate the target item prediction offset values when different attack profiles are injected while the attack sizes and filler attacks vary.

**Fig 2:**
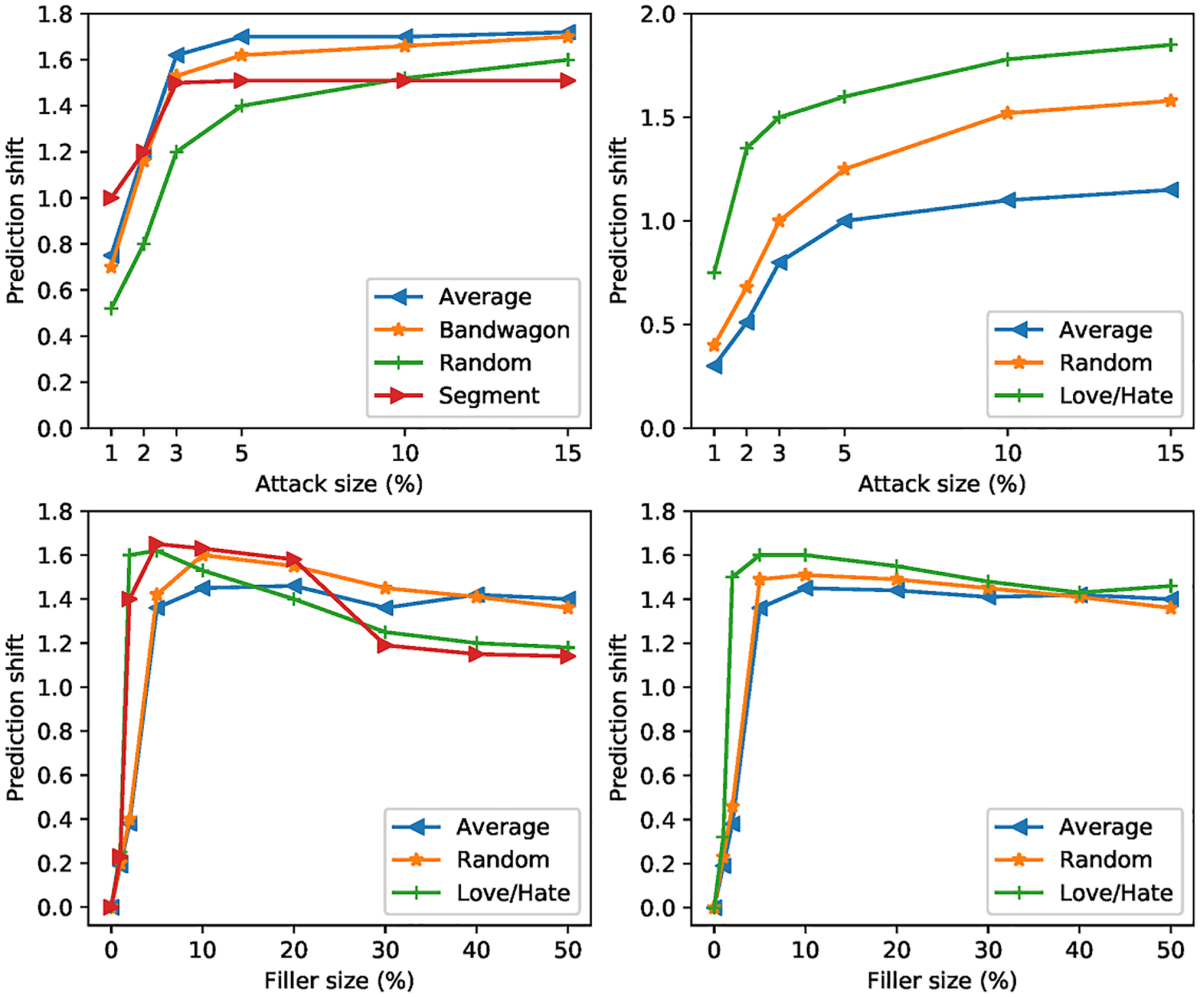
Prediction shift in user-based collaborative filtering with attack size varies and filler size varies.

From Fig 2, we can see that the value of prediction shift becomes greater when attack size increases. When the filler size increases, after reaching an extreme value, the value of prediction shift decreases. We can infer from the experiment that the value of prediction shift does not simply increase with filler size and attack size increase. To obtain the proper prediction shift, attackers artificially generate forged profiles with unfixed filler and attack sizes in a specific period of time.

## 4 Shilling attack detecting approach based on a two-phase structure

A shilling attack detection method is proposed based on the group user credibility prediction model. First, trust prediction model of group users will be analysed. then, a time series of ratings are proposed to detect suspicious time windows of ratings. Finally, target item analysis method is used to filter shilling attacks. To obtain a considerable prediction shift of the target item in the recommendation list, attackers use a set of profiles to perform the attack instead of just a single profile. A certain number of attack profiles can alter the target item’s recommendation list. Attack profiles can be considered as a group instead of a single profile.

**Fig 3:**
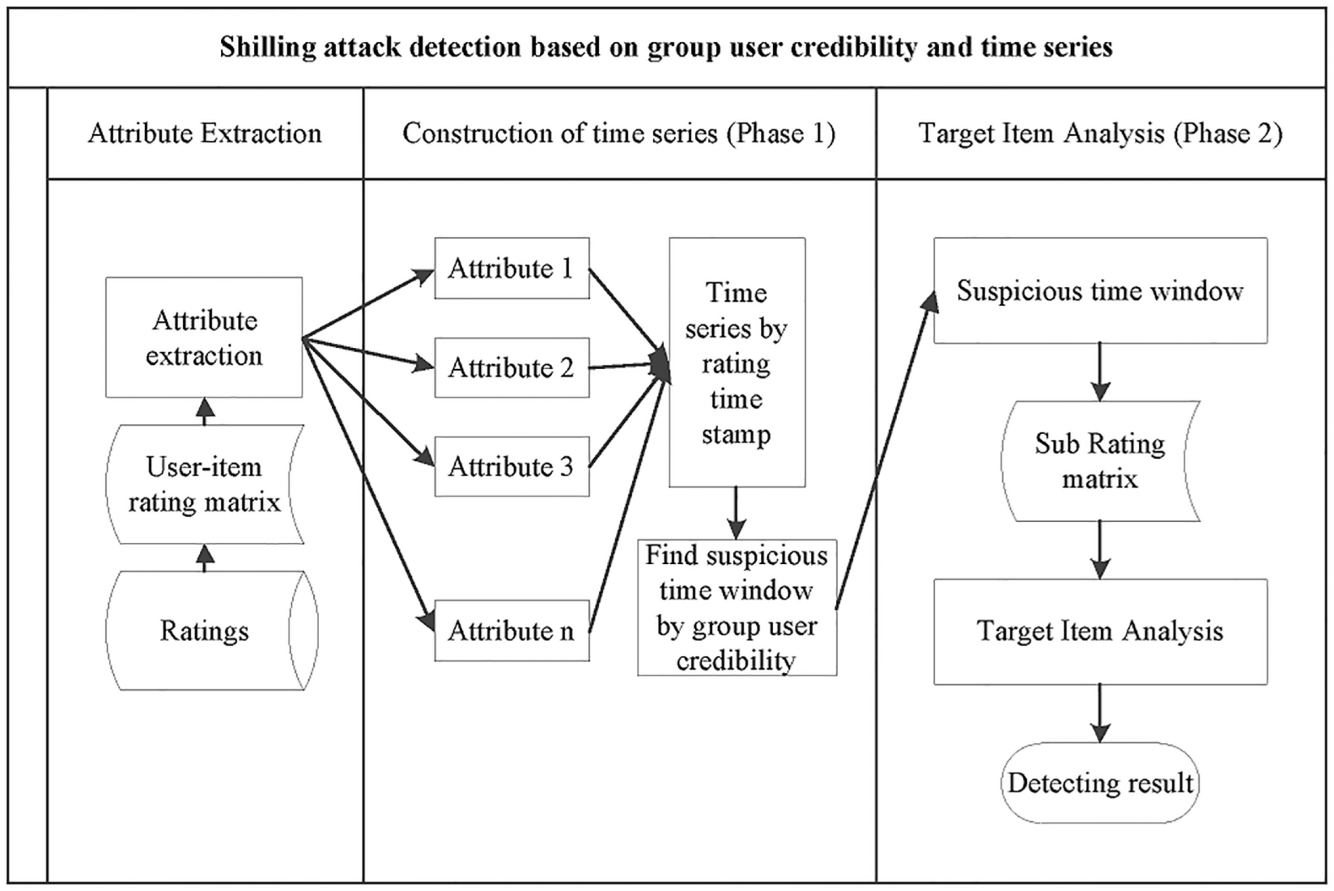
Overall structure of the proposed shilling detecting method.

In this paper, a two phases shilling detecting structure is proposed. Fig 3 is the overall structure of the method.

In the first stage, all ratings of the project are ranked by a rating time stamp for each project. The attributes of the ratings and profiles are extracted, and a data stream based on the attributes is constructed. A rough result containing the attack profile and the real profile is obtained in this stage. Time intervals at which the attack profile is injected into the system are divided. The second phase is a fine-tuning stage that filter genuine profile from attack profiles by target item analysis method. At this stage, reducing the genuine profile in the final detecting result based on sparseness characteristics, which can reduce the misjudgment rate of the final result. Target item analysis method is described in [21].

### 4.1 Credibility of group users by rating prediction model

In this section, a trust model based on rating variances is proposed to calculate the overall credibility of all user profiles and use the credibility as a descriptor to determine the likelihood of being shilling attackers in each time interval. The assumption is that there exist differences between forged ratings and ratings of genuine profiles. The rating variances of all ratings of an attack profile should be higher than the rating variances of all ratings of a genuine profile. The accuracy of predictions decreases with the increase of rating variance, and as a result, rating variance is considered a confidence measurement descriptor related to recommender systems [22].

*credibility score* *q*_*i*_ is proposed for every user(profile) *p* based on all the ratings, denoted as *r_u,i_*′, as well as the item-specific rating variance. *R*_*u*, *i*_ is the set of ratings of the profile *p* from user-item rating matrix, while predicted rating *r_u,i_*′ is the predicted rating made by the user-item rating matrix. The following equation illustrates a simple method:

$$\begin{array}{c} q_{i}=\frac{\sum_{u=1}^{\left|u\right|}{r_{u,i}}^{\prime }v_{u,i}}{\sum_{u=1}^{\left|u\right|}v_{u,i}} \end{array}$$ (11)

where *v*_*u*, *i*_ is a rating-variance-based vote defined as:

$$\begin{array}{c} v_{u,i}=\{{}_{0,\;\;\;\;\;\;|R_{u,i}-q_{i}|>\Delta }^{1\;\;\;\;\;\;|R_{u,i}-q_{i}|\leq \Delta } \end{array}$$ (12)

A rating deviation threshold Δ is set, which is the maximum acceptable rating variance for any trust-based rating predication. The method used for setting the threshold value depends on the dataset used, chosen from the suggested range, or defined as the worst-case standard deviation. When the threshold value Δ is set as a large value, some forged profiles have a high probability of being considered normal profiles. However, if the threshold value Δ is set to be a small value, some genuine profiles would be considered as attack profiles. Based on experience, the threshold value is set in a range from 0.8 to 1.2 in hybrid recommendation systems.

As we have introduced in Section 1, filler items of attack profiles are generated according to the average score of the rated items. The rating score may be close to the mean score of the rated items, but the rating variance score of the profile will be greater than that of genuine profiles. We noted that rating deviation was an important attribute to detect shilling attacks; the attribute can be used to separate shilling attack profiles from normal profiles, especially to detect profiles with extreme ratings. Profiles that with high rating variance scores are likely to be attack profiles, which are then marked as suspicious shilling attack profiles. In the next section, rating variance and other attributes are used to construct a data stream by rating timestamps.

**Fig 4:**
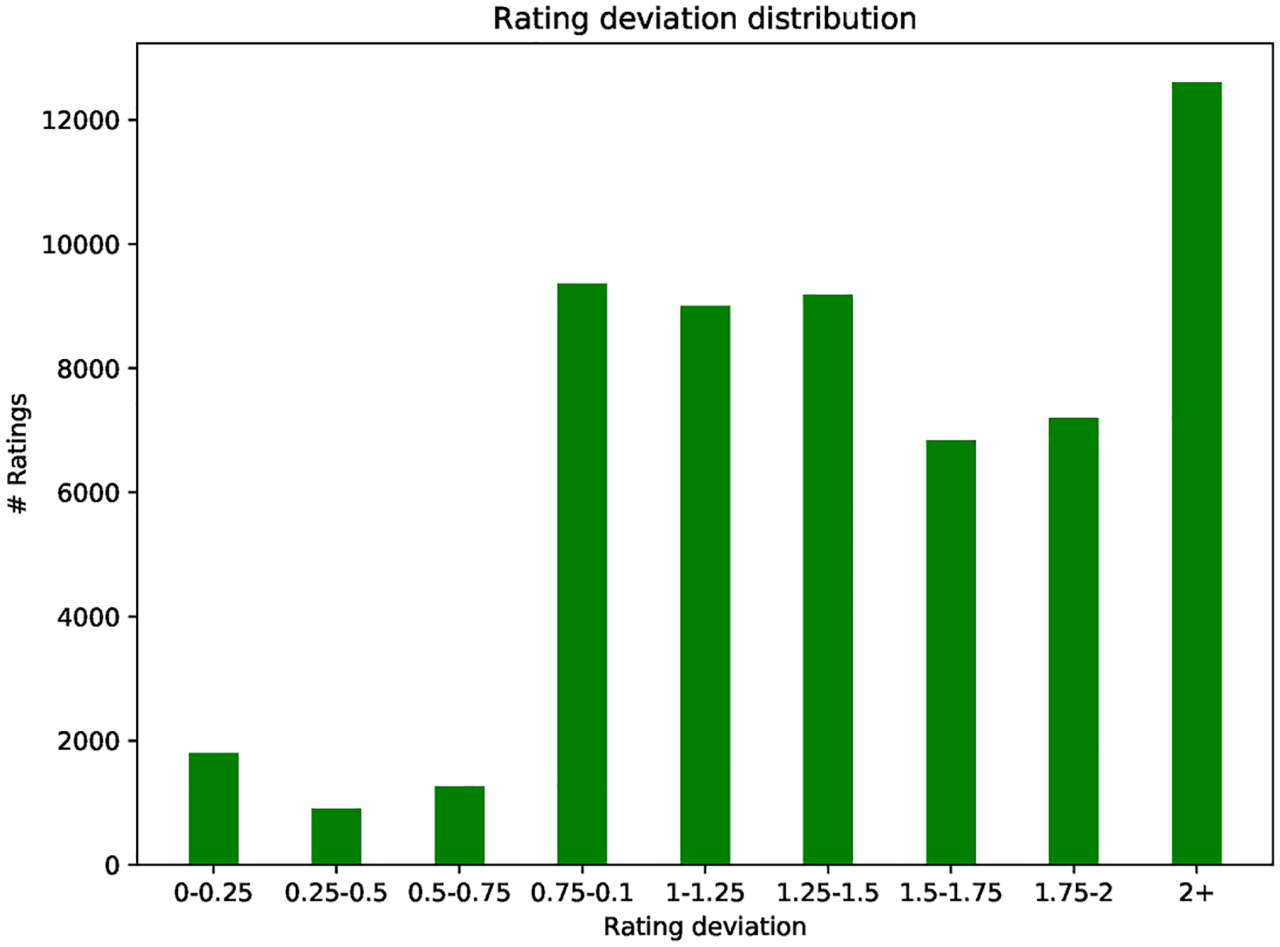
Rating deviation distribution.

Fig 4 shows the rating deviation distribution of MovieLens 1M ratings dataset using standard user-based CF methods. We can see in Fig 4 that a large number of predicated ratings fall within the range of small deviations (less than 2) for all ratings in the dataset. This means that the difference between the ratings of most normal ratings is very small. If an abnormal rating variance occurs in a profile for a period of time, the profiles are considered to be abnormal, which provides support for group users to detect the credibility of anomalous profiles.

### 4.2 Locate suspicious time intervals by rating time series

It is time-consuming to perform machine learning methods in large datasets. There are too many items and user profiles in real-time recommendation system, so it is time-consuming to perform detection on the entire system. Most user profiles in the recommender systems are not suspicious of being attack profiles. The entire dataset is divided into a subset of subsections, and each subsection is examined separately. The techniques presented in previous studies were used to focus on the user-rating matrix of suspicious profiles. The target dataset will be greatly reduced in search scope, which will save time and increase efficiency.

There are two stages in the detection structure. In the first stage, we found suspicious ratings for each item and then found a segmented suspicious rating of the item at a particular time. Suspicious rating segments are determined by constructing a time series. In this stage, the number of profiles is reduced by a large margin. We then use statistical measures and target item analysis to detect the anomalous ratings in the first phase. Abnormal rating section will be analysed and misjudged genuine profiles would be filtered out. All ratings of an item are classified into data streams by time stamps, and then classify *w* (time window) consecutive ratings into a window. According to the attributes of abnormal group characteristics, a multi-dimensional time series can be selected, including the number of ratings per unit time, the average rating and frequency of comments. For example, in online stores, items are sorted in ascending time stamps, which gives

$$\begin{array}{c} R(s)=\{r_{1},r_{2},r_{3},\cdots ,r_{i},\cdots ,r_{j},\cdots ,r_{n_{s}}\} \end{array}$$ (13)

$$\begin{array}{c} TS(s)=\{t_{s_{1}},t_{s_{2}},t_{s_{3}},\cdots ,t_{s_{i}},\cdots ,t_{s_{j}},\cdots ,t_{s_{n}}\} \end{array}$$ (14)

where *R*(*s*) is a series of ratings users who rated the item and *TS*(*s*) is the time series of the item’s ratings. For all ratings, 1 ≤ *i* ≤ *j* ≤ *n*, *t_s_i__* ≤ *t_s_j__*. For example, *t_s_i__* is the timestamp of rating *r*_*i*_.

A time window Δ*t* is used to divide the rating time interval *I* = [*t*_0_, *t*_0_ + *T*] into *n* = *T*/Δ*t* time windows, where the length of every time window is Δ*t*, and *t*_0_ is the starting time stamps. For the *i*th time window *I*_*i*_, *I*_*i*_ = [*t*_0_ + (*i* − 1) Δ*t*, *t*_0_ + *it*_0_], where $I=\overset{n}{\underset{i=1}{\cup }}I_{i}$. For each time window in the time series *I*_*i*_, the eigenvalue is calculated. Thus, in a user-rating dataset, given time interval [*t*_0_, *t*_0_ + *T*] and the time window Δ*t*, a time series can be derived:

$$\begin{array}{c} F_{s}(I,\Delta t)=[\begin{array}{c} I_{1}(1),I_{1}(2),\cdots ,I_{1}(i),\cdots ,I_{1}(j),\cdots \;I_{1}(n)\\ I_{2}(1),I_{2}(2),\cdots ,I_{1}(i),\cdots ,I_{1}(j),\cdots \;I_{2}(n)\\ I_{3}(1),I_{3}(2),\cdots ,I_{1}(i),\cdots ,I_{1}(j),\cdots \;I_{3}(n) \end{array}] \end{array}$$ (15)

Abnormal user discovery based on multidimensional time series can reveal the group attributes of abnormal users. If the multidimensional feature is abnormal in the time series, all ratings in the time interval are recognized as exceptions. A set of suspicious ratings can be found in this step. There are false positives in the suspicious ratings, so it is necessary to filter normal ratings from the suspicious ratings.

## 5 Experiments

In this section, we conducted a wide range of experiments on different datasets and benchmarking methods. The experimental results of the shilling attack detection based on group user credibility and time series are introduced and discussed.

### 5.1 Experiment setup

The MovieLens datasets were published by GroupLens at the University of Minnesota, are mainly used in the experiments. In addition to MovieLens datasets, a subset of Netflix dataset and subset of Eachmovie dataset are also used. A rating score of 0 means that there are no user ratings for the corresponding item. We have filtered out users who have rated fewer than 20 items.

### 5.2 Evaluation metrics

Indicators like *Detection Rate*, *False Positive rate* were used to evaluate the proposed method. *Attacks* is the number of attacks while *Detection* is the number of detected profiles.

$$\begin{array}{c} Detection\;Rate=\frac{\#Detection}{\#Attacks} \end{array}$$ (16)

*FalsePositives* is the number of misjudged genuine profiles while *GenuineProfiles* is the number of genuine profiles.

$$\begin{array}{c} False\;Positive\;Rate=\frac{\#False\;Positives}{\#Genuine\;Profiles} \end{array}$$ (17)

### 5.3 Locate suspicious attack segments by rating time series

Attack profiles are generated according to the composition of different attack models. Two parameter, attack size and filler size are varied in the experiment. In order to get a certain prediction shift, attack sizes varies from 2% to 14%, and filler size varies from 1% to 10%. ∂ is defined as follows:

$$\begin{array}{c} \partial =\frac{a}{A} \end{array}$$ (18)

In this equation, *a* indicates attack profiles falls into a suspicious window, and *A* represents the number of attack profiles injected.

When the confidence coefficient changes, Table 2 shows the change in ∂. In Table 2, the value of ∂ depends on the confidence coefficient and attack size. In general, the ∂ value increases when the confidence coefficient increases. The false positive rate will increase when larger confidence coefficients are chose. In this situation, genuine profiles can be filtered at the second phase. However, the false negative rate will increase when lower confidence coefficients are chose. When an attack profile is misjudged as a genuine profile, it can not be retrieved at the second phase. Taken together, a 90% confidence coefficient was selected in this paper. The percentage of attack profiles that fall within the suspicious time window increases as the attack size increases. Attack profiles that fall within suspicious time windows increases as the filler size of attack profiles increases.

**Table 2:** Attackers in suspicious rating segments ratio in phase 1 when attack size and confidence coefficient vary.

confidence coefficient	Attack size									
	2%	4%	6%	8%	10%	12%	14%	16%	18%	20%
80%	0.895	0.915	0.935	0.942	0.95	0.955	0.955	0.96	0.965	0.97
85%	0.90	0.919	0.941	0.945	0.954	0.957	0.96	0.963	0.965	0.971
90%	0.91	0.93	0.945	0.948	0.958	0.962	0.965	0.967	0.97	0.972
95%	0.92	0.931	0.945	0.952	0.96	0.965	0.966	0.965	0.97	0.971

Table 3 shows the detection rate when the attack size changes at a confidence factor of 90%. From the table we can indicate that the detection rate increases with attack size. When the attack size is less than 10%, the detection rate with larger filler size higher than the detection rate with lower filler size as the attack size is the same. False positive rate increases as attack size increases.

**Table 3:** Attack detection ratio when attack size varies under confidence coefficient 90%.

Filler size (%)	Attack size (%)									
	2%	4%	6%	8%	10%	12%	14%	16%	18%	20%
3%	0.69	0.74	0.79	0.83	0.865	0.89	0.90	0.91	0.91	0.913
5%	0.74	0.78	0.82	0.86	0.89	0.905	0.916	0.925	0.93	0.941
7%	0.78	0.83	0.87	0.895	0.91	0.92	0.928	0.942	0.946	0.95
9%	0.81	0.85	0.88	0.905	0.92	0.935	0.945	0.95	0.954	0.955

### 5.4 Shilling attack detection based on time series and group user’s confidence

In this section, shilling detection method based on rating time series analysis and group users’ credibility is examined. In the experiments, attack size varies from 2% to 14%; filler size varies from 3% to 9%.

**Fig 5:**
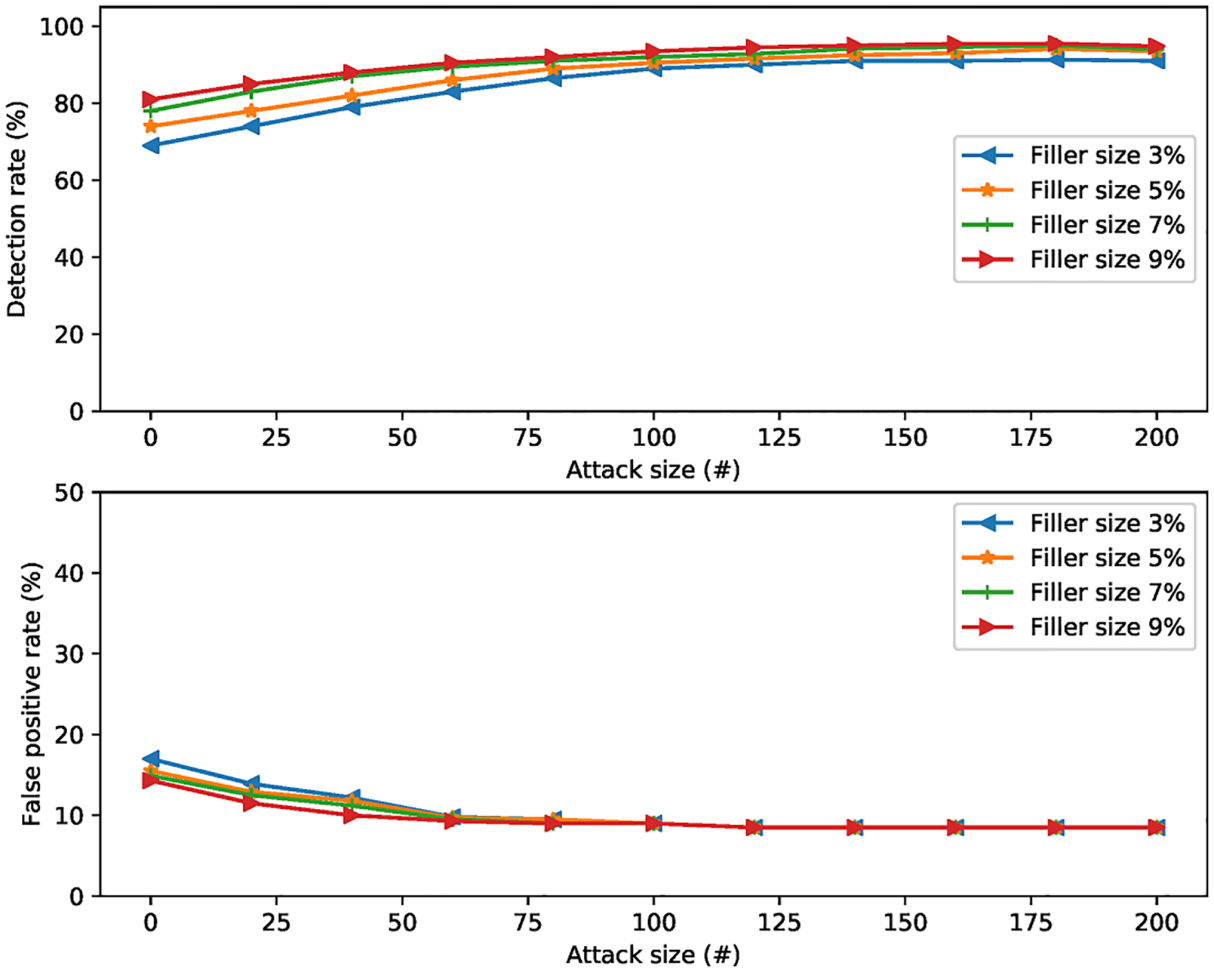
Detection rate and false positive rate when attack size varies.

Fig 5 shows detection rate and false positive rate of different target item analysis based algorithms. We have tried four ranges of filler sizes: 3%, 5%, 7%, 9%. From the figure we can see that the detection rate is very low in all cases with a lower attack and filler size. The detection rate increases as the filler size increases. When the number of attack size is greater than 100, the detection rate is more than 90%.

**Fig 6:**
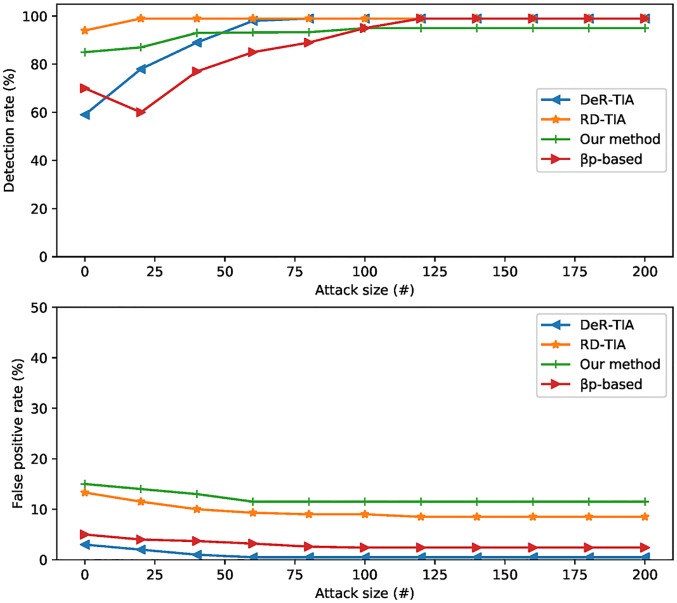
Comparisons of detection results with other methods.

Fig 6 shows the comparisons of detection results using different detection methods. We employed state-of-the-art shilling detection methods [23] and other detection methods that we have proposed [24]. *βρ*-based method gets better detection rate than other *TIA*-based methods. False positive rate of our method is higher than that of *DeR-TIA* and *RD-TIA*. *βρ*-based method has the highest false positive rate. The detection rate of four algorithms becomes higher when attack size increases.

**Fig 7:**
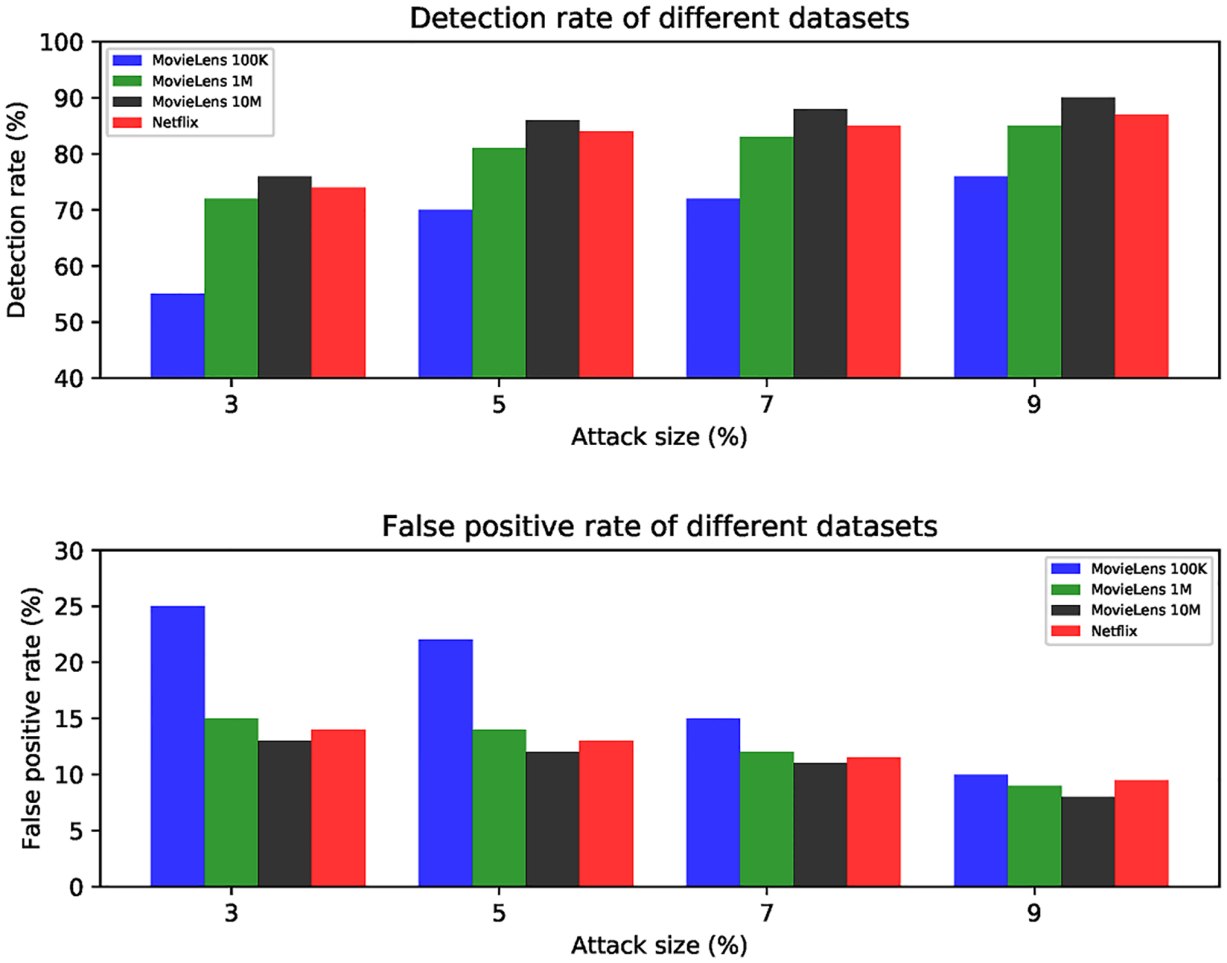
Detection results with different datasets with attack size varies.

Fig 7 shows the detection result of different dataset. There are four datasets are used in the experiment, MovieLens 100K dataset, MovieLens 1M dataset, subset of the Netflix dataset and MovieLens 10M dataset. The rating matrixes of these four datasets are incremental. We can see from Fig 7 that the detection rate increase when the attack size increases. From the dataset view, the detection rate is higher when using larger datasets. This is because the number of attack profiles gets greater when the rating matrix increases. From the attack size view, the false positive rate decreases when the attack size increases. Conversely, the false positive rate is higher when detecting a smaller dataset. Overall, the proposed method performs better when the attack size is greater.

## 6 Conclusion and future work

In this paper, a shilling detection method for user-based recommender systems based on credibility of group users and rating time series is proposed. The idea is built on the assumption that the credibility of group users has a negative correlation with rating variance. Considering group and timeliness features of shilling attacks, a credibility evaluation model based on rating prediction model to derive proximity-based predictions is proposed. Suspicious rating time segments are determined by constructing a time series for each item, and then data streams are examined and suspected time windows are checked.

In analyzing features of shilling attacks by group users, abnormal group user discovery method based on time series and time window is proposed. We test the method on different datasets and actual data in e-commerce to verify the model and algorithm. The experimental results show that the proposed method performs well when detecting shilling attacks within a small-time window. The time-consumption of the proposed method increases linearly with the increase of scale of datasets, while traditional detection methods cannot achieve such an effect. In the group credibility model, only the rating factor between users is considered. In the future, additional factors will be considered, including more user behaviours such as personal information and social relationship. We will continue to examine the issue of shilling detection from two dimensions, time series and credibility of group users. When calculating the credibility of group users, other factors will be used, such as social relationships, user preferences (like or dislike) will be considered.
